# The colours and contours of compassion: A systematic review of the perspectives of compassion among ethnically diverse patients and healthcare providers

**DOI:** 10.1371/journal.pone.0197261

**Published:** 2018-05-17

**Authors:** Pavneet Singh, Kathryn King-Shier, Shane Sinclair

**Affiliations:** 1 Faculty of Nursing, University of Calgary, Calgary, Alberta, Canada; 2 Department of Community Health Sciences, Cumming School of Medicine, University of Calgary, Calgary, Alberta, Canada; 3 Department of Oncology, Cumming School of Medicine, University of Calgary, Calgary, Alberta, Canada; University Antwerp, BELGIUM

## Abstract

**Objective:**

To identify and describe the perspectives, experiences, importance, and impact of compassionate care among ethnically diverse population groups.

**Methods:**

A systematic search of peer-reviewed research focused on compassionate care in ethnically diverse populations published between 1946 and 2017 was conducted.

**Results:**

A total of 2296 abstracts were retrieved, out of which 23 articles met the inclusion criteria. Synthesis of the literature identified the perspectives, facilitators and barriers of compassion in healthcare within ethnic groups. Compassion was described as being comprised of healthcare provider (HCP) virtues (honesty, kindness, helpful, non-judgment) and actions (smile, touch, care, support, flexibility) aimed at relieving the suffering of patients. The importance and impact of providing compassion to ethnically diverse patients was also identified which included overcoming cultural differences, alleviating distress at end-of-life, promoting patient dignity and improving patient care. This review also identified the need for more contextual studies directly exploring the topic of compassion from the perspectives of individuals within diverse ethnic groups, rather than superimposing a pre-defined, enculturated and researcher-based definition of compassion.

**Conclusions:**

This review synthesizes the current evidence related to perceptions of compassion in healthcare among diverse ethnic groups and the role that compassion can play in bridging ethno-cultural differences and associated challenges, along with identifying gaps in literature related to compassionate care within diverse ethnic groups. Establishing an evidence base grounded in the direct accounts of members of diverse ethnic communities can enhance culturally sensitive compassionate care and improve compassion related health outcomes among diverse ethnic groups.

## Introduction

Western countries are increasingly becoming ethnically and culturally diverse. For example, ethnic minorities represent 27% of the population in Canada [1], 26% in the USA [2], and 13% in the UK [3]. As a result, Western healthcare systems are serving a larger number of ethnically diverse patient populations, thus signifying the need for providing culturally competent patient care [4–6]. Cultural competence is considered imperative to provide quality care to patients from diverse ethnic backgrounds [7–9]. Ethnicity primarily refers a group of individuals who are connected by racial, geographic origins, language and other shared traits whereas culture refers to a group of individuals, within or outside of an ethnic group, who are connected by social customs, norms and institutions [10, 11]. Ethnicity and culture are often intertwined as people from different ethnicities often have differences in cultural practices [12], emphasizing the need for providing culturally competent care to patients from different ethnicities. A review summarizing the cultural barriers in healthcare identified the following: socioeconomic, racial, and ethnic disparities in accessing healthcare services; lack of management of the healthcare needs of immigrant patients; need for healthcare providers (HCPs) to be culturally sensitive and avoid stereotyping; and the importance of HCP recognizing similarities and differences between and how to integrate them into clinical practice [13].

Compassion is considered a key characteristic of quality healthcare by patients and their families, HCPs, and policy makers [14–19], with a potential to promote greater respect for patient’s dignity [20]. It has been defined in healthcare literature as “a virtuous response that seeks to address the suffering and needs of a person through relational understanding and action” (Page 195) [21]. The importance and experiences of compassion are considered to be cross-cultural, while also spanning spiritual and religious traditions [22–24]. The Charter for Compassion urges cultures and religions of the world to embrace compassion as a core value, noting that “The principle of compassion lies at the heart of all religious, ethical and spiritual traditions, calling us always to treat all others as we wish to be treated ourselves” [25]. While the universality of compassion and its function as a medium for culturally sensitive care is presumed, a systematic evaluation of the literature to validate these claims is needed.

Increasingly, compassion is regarded as the foundation of patient-centered care, however there is a lack of research to determine the transferability of a patient centered approach to ethnically diverse populations and the relationship of compassion to this end [21, 26, 27]. A recent scoping review describing compassion in healthcare identified that the vast majority of studies have been conducted in Western settings, significantly limiting the generalizability of the findings [27]. The authors recommended that studies be conducted on the topic with participants from different ethnicities in order to determine if compassion varies across different ethnic groups [27]. Furthermore, while compassion seems to be important across healthcare, it is extremely important to both patients and their families, who are facing the end of life [28–32]. Considering how death and dying; end of life rituals, customs, and spiritual needs vary among different ethnicities [33, 34], recognizing ethnic variances of compassion and providing culturally sensitive compassionate care at end of life is particularly important. Since compassion is a highly relational care construct, involving the personhood of both the patient and the HCP, ethnic differences may inadvertently act as barriers to receiving or providing compassionate care, whereby the good intentions of the HCP do not have their intended effect [35]. For example, in Western cultures, honesty and truth-telling (e.g., disclosing prognostic information directly to patients), are often considered hallmarks of quality care in general and compassionate care specifically, whereas this approach may have the opposite effect in other cultures that believe protecting patients from the truth is a hallmark of compassionate care [35, 36]. In addition, cultural and language differences between HCP and patients can serve as barriers to providing quality healthcare in general [13, 37]. Patients of different ethnicities could unintentionally perceive certain actions of HCPs as non-compassionate. For example, even though supportive touch is generally considered an act of compassion across various cultures, it can be perceived as non-compassionate in certain cultures if the HCP and the patient are of a different gender [38]. Therefore, expanding the understanding of how different ethnic groups understand and experience compassion is important in informing HCPs working within ethnically diverse groups.

In this review, we describe the perspectives, importance, experiences, and facilitators/barriers of compassion within healthcare among diverse ethnic patient populations in order to highlight the current evidence about ethnic variances of compassion and identify gaps in literature.

## Methods

### Study design

A systematic review, in accordance with a narrative synthesis approach was used. Narrative synthesis is a textual approach that appraises the research based evidence of a large and diverse literature base by ‘telling the story’ to explain the findings [39]. This approach focuses on an interpretive synthesis of findings rather than a meta-analysis of data or evaluating the quality of evidence based strictly on statistical analysis, a recommended approach when the topic of interest is emerging, abstract, and/or poorly defined.

### Search methods

The following databases were searched: MEDLINE, Cumulative Index to Nursing and Allied Health Literature (CINAHL), EMBASE, and PsycInfo. Key terms included compassion, culture, ethnicity, and healthcare. The search was conducted in May 2017, and updated in October 2017 (see Table 1 for MEDLINE search strategy). Initial screening was undertaken by two members of the research team reviewing title and abstracts (PS, SS) to ensure that the retrieved citations were focused on compassionate care among ethnically diverse populations. Full texts of the articles were further screened in a similar process using the inclusion criteria: (1) published in English; (2) quantitative or qualitative research, review articles, or opinion pieces; (3) related to compassion in healthcare; and (4) focused on ethnically diverse populations (Table 2). Articles that were not written in English, which were not focused on healthcare, did not include ethnically diverse populations, and which were focused on empathy or sympathy were excluded. The reference lists of the retrieved articles were also manually searched to identify any additional relevant articles. Full-text articles were reviewed by two members of the research team (PS, SS) with another member of the research team (KKS) providing direction and settling disputes as needed.

**Table 1:** Ovid MEDLINE(R) (1946 to 2017) search strategy.

#	Searches	Results
1	exp Research Subjects/	14029
2	exp Research/	549523
3	exp Research Design/	396792
4	exp Research Report/	2336
5	exp Qualitative Research/	33585
6	research stud*.mp.	17799
7	1 or 2 or 3 or 4 or 5 or 6	864863
8	exp Ethnic Groups/	136380
9	exp "Emigrants and Immigrants"/	8933
10	exp Minority Groups/	12179
11	exp Minority Health/	608
12	exp Cultural Diversity/	10650
13	exp Culture/	147186
14	multi-ethnic*.mp.	3110
15	multiethnic*.mp.	3662
16	ethnic*.mp.	152212
17	racial*.mp.	35100
18	minority.mp.	56756
19	minorities.mp.	9610
20	"ethnic/racial".mp.	812
21	"racial/ethnic".mp.	8579
22	immigrant*.mp.	24777
23	asian*.mp.	116394
24	chinese.mp.	190312
25	korea*.mp.	63348
26	japan*.mp.	224934
27	india*.mp.	183398
28	mexico*.mp.	65163
29	hispanic*.mp.	47525
30	latino*.mp.	10174
31	indian*.mp.	86022
32	china*.mp.	173981
33	iran*.mp.	38654
34	philippin*.mp.	11660
35	africa*.mp.	267676
36	pakistan*.mp.	20386
37	indigenous.mp.	26589
38	latina*.mp.	3466
39	native*.mp.	185678
40	filipino*.mp.	2723
41	exp Asian Continental Ancestry Group/	59506
42	exp African Continental Ancestry Group/	78618
43	exp African Americans/	47755
44	exp Hispanic Americans/	27491
45	exp Indians, North American/	13545
46	exp Oceanic Ancestry Group/	8659
47	exp Arabs/	3736
48	cultur*.mp.	1571113
49	exp Cultural Characteristics/	15532
50	8 or 9 or 10 or 11 or 12 or 13 or 14 or 15 or 16 or 17 or 18 or 19 or 20 or 21 or 22 or 23 or 24 or 25 or 26 or 27 or 28 or 29 or 30 or 31 or 32 or 33 or 34 or 35 or 36 or 37 or 38 or 39 or 40 or 41 or 42 or 43 or 44 or 45 or 46 or 47 or 48 or 49	2990248
51	compassion*.mp.	7639
52	compassionate care*.mp.	688
53	compassionate*.mp.	3973
54	51 or 52 or 53	7639
55	exp "Delivery of Health Care"/	946746
56	healthcare*.mp.	166084
57	exp Health Services/	1874341
58	exp Hospitals/	247254
59	clinic*.mp.	4108723
60	clinical*.mp.	3777057
61	medical system*.mp.	5695
62	exp Nursing/	243484
63	exp Nursing Care/	128519
64	exp Nursing Research/	51564
65	healthcare system*.mp.	16256
66	exp Physicians/	115068
67	exp Nurses/	80849
68	exp Patients/	55014
69	55 or 56 or 57 or 58 or 59 or 60 or 61 or 62 or 63 or 64 or 65 or 66 or 67 or 68	6294558
70	7 and 50 and 54 and 69	109
71	50 and 54 and 69	775
72	limit 71 to english language	751
73	limit 72 to (addresses or autobiography or bibliography or biography or case reports or clinical conference or comment or congresses or consensus development conference or consensus development conference, nih or dataset or dictionary or directory or editorial or guideline or interactive tutorial or interview or lectures or legal cases or legislation or letter or news or newspaper article or overall or periodical index or personal narratives or portraits or video-audio media or webcasts)	74
74	72 not 73	677

* retrieves all suffix variants of a search term

**Table 2:** Inclusion criteria.

1	Language	Published in English
2	Research design	Empirical research (quantitative, qualitative or mixed methods), review articles, or commentaries
3	Participants	Study participants included ethnically diverse populations (e.g., Asians, Hispanics, African Americans, Native Americans)
4	Study focus	Related to compassion or compassionate care in healthcare

### Quality appraisal

Each article was appraised independently (by PS and SS) using the Mixed Methods Appraisal Tool (MMAT) by Pace et al. [40]. MMAT (http://mixedmethodsappraisaltoolpublic.pbworks.com) uses a simple checklist approach to examine the methodological quality of research studies (quantitative, qualitative, and mixed methods studies), but not of non-research articles (e.g. review articles, commentaries). The appraisal included examination of data sources and selection/sampling/response rate; as well as data collection and analysis. There was 91% inter-rater reliability between each reviewers’ independent appraisals of the full articles. Differences in appraisals were resolved after discussion between the reviewers (PS, SS) with disputes being resolved by a third researcher (KS) (see Table 3 for MMAT appraisal of articles). Articles were not excluded on the basis of their quality in order to include all relevant articles.

**Table 3:** Data extraction and evaluation of studies.

Author	Year	Journal	MMAT Score	Study Country	Study Population(s)
Arthur, D. et al.	1998	Contemporary Nurse	****	Hong Kong	Chinese
Babaei, S. et al.	2016	International Nursing Review	**	Iran	Iranian
Baker, C. et al.	2000	Western Journal of Nursing Research	***	Canada	First nations
Bosma, H. et al.	2010	Palliative Medicine	N/A (review article)	Canada	N/A (review article)
Brooten, D. et al.	2013	American Journal of Hospice & Palliative Medicine	*	USA	Hispanic, Black, White
Chang, D.F. et al.	2011	Psychotherapy Research	****	USA	Asian, African-American, Hispanic, multiracial
Fox, H. et al.	2015	Journal of Pain & Palliative Care Pharmacotherapy	N/A (narrative)	India	N/A (narrative)
Garrett, P.W. et al.	2008	Ethnicity & Health	*	Australia	Arabic, Italian, Vietnamese, Chinese, Croatian, Serbian, Spanish
Jenkins	2005	Journal of Palliative Medicine	**	USA	African American
Kim, S. et al.	2007	Issues in Mental Health Nursing	N/A (commentary)	South Korea, USA	N/A (commentary article)
Kongsuwan, W. et al.	2011	Intensive and Critical Care Nursing	***	Thailand	Thai
Kwan, M.L. et al.	2013	Breast Cancer Research and Treatment	****	USA	White, Asian, African-Americans and Hispanics
Lori, J.R. et al.	2011	Journal of Transcultural Nursing	**	USA	African-Americans
Lundberg, P.C. et al.	2001	Journal of Advanced Nursing	***	Thailand	Thai
Manookian, A. et al.	2014	Nursing Ethics	**	Iran	Iranian
Msiska, G. et al.	2014	Nurse Education Today	***	Malawi	African
Napoles, A.M. et al.	2009	Health Services Research	***	USA	African-American, Latinos, White
Papadopoulos, I. et al.	2016	International Nursing Review	****	UK	Participants from Australia, Cyprus, Czech Republic, Greece, Hungary, Italy, Israel, Norway, Philippines, Poland, Colombia, Spain, Turkey, UK and USA
Papadopoulos, I. et al.	2015	Journal of Transcultural Nursing	****	UK	Participants from Australia, Cyprus, Czech Republic, Greece, Hungary, Italy, Israel, Norway, Philippines, Poland, Colombia, Spain, Turkey, UK and USA
Sethabouppha, H. et al.	2005	Archives of Psychiatric Nursing	**	Thailand	Thai
Shimoinaba, K. et al.	2014	Palliative & Supportive Care	***	Japan	Japanese
Valizadeh, L. et al.	2016	Nursing Ethics	****	Iran	Iranian
Zamanzadeh, V. et al.	2017	Scandinavian Journal of Caring Sciences	****	Iran	Iranian

Each article was appraised using the Mixed Methods Appraisal Tool (MMAT) which uses an easy checklist approach to examine qualitative, quantitative, and mixed methods studies ^37^.

Scores vary from 25% (*, one criterion met),

50% (**, two criterion met),

75% (***, three criterion met),

to 100% (****, all criteria met).

## Results

### Search outcomes

**Fig 1:**
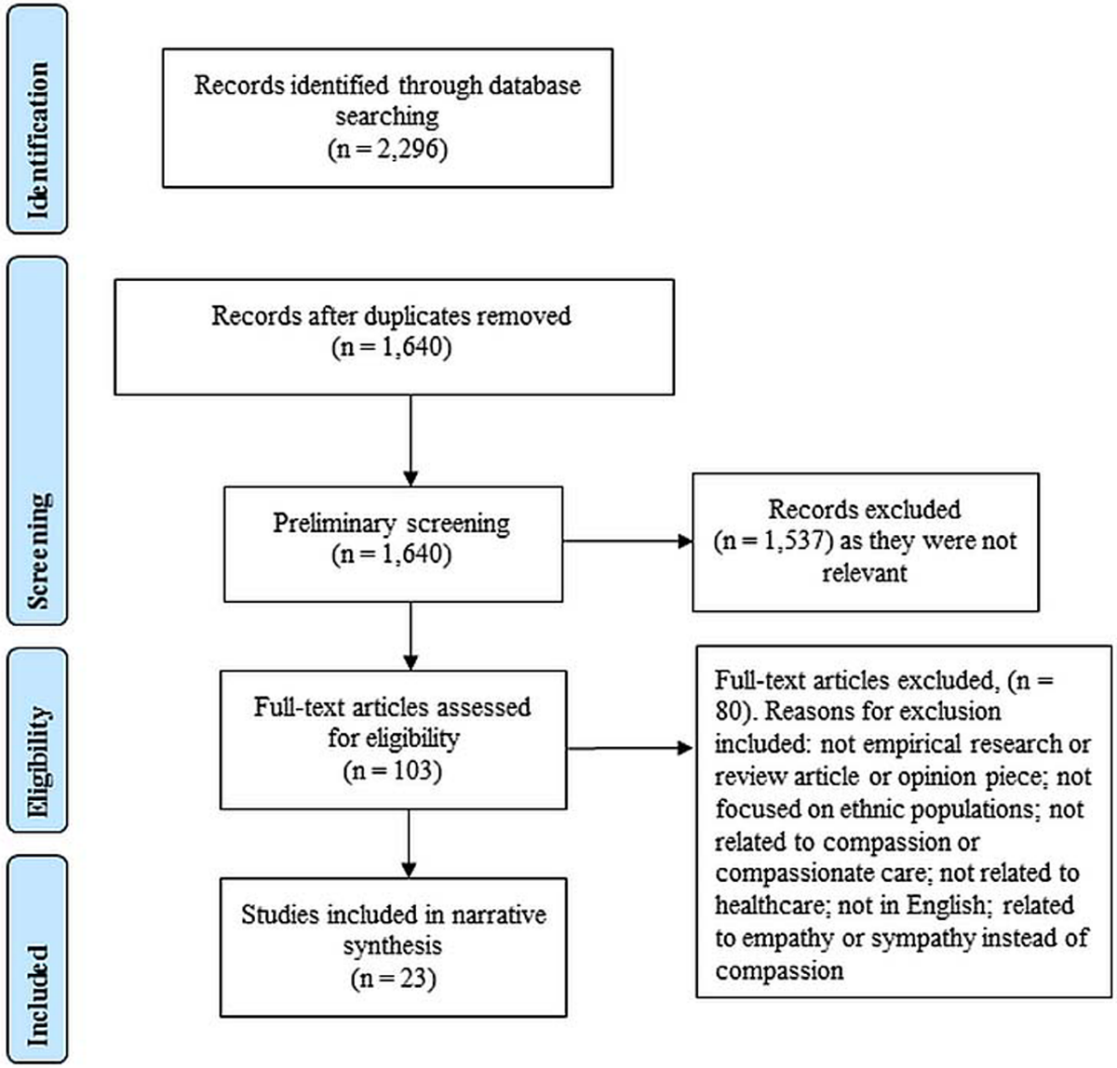
Search and selection process.

The search strategy yielded 2296 results. Duplicates were removed and title and abstract screening was undertaken, rendering 103 results. The full-texts of these articles were then retrieved and screened using the aforementioned inclusion criteria. The reference list of these articles was also searched to identify any additional relevant articles. As a result of this process, 23 articles met the inclusion criteria and were included in this review. The search outcome, illustrated in Fig 1, generated three overarching categories: (1) perspectives on compassion in healthcare; (2) perceptions on facilitators and barriers to compassion; and (3) perspectives on the importance and impact of providing compassion to ethnically diverse patients.

### Perspectives on compassion in healthcare within ethnic groups

Several authors investigated the nature of compassion from the perspective of ethnically diverse HCPs and patients’ groups, with the majority of these authors utilizing an apriori definition of compassion versus a definition generated by members of these ethnic groups. Papadopoulos et al. conducted a study to explore the similarities and differences in the understanding and demonstration of compassion in nurses from 15 countries using online surveys [41]. They reported that there was generally a shared understanding of compassion across cultures which was defined as a “deep awareness of the suffering of others and a wish to alleviate it” (Page 289), and involved actions including giving time, being there, and getting to know the patient [41]. While the study did mention a few differences in the perceptions of compassion among nurses from different countries, they were not elaborated on, nor clearly delineated. Additionally, while the ethnic background of individual participants was not reported, 66% of survey respondents were currently working in Western countries, thus limiting the generalizability of the findings of this study across diverse ethnic groups. Papadopoulos et al. also conducted an international survey on nurses’ views and experiences of compassion [42]. Study participants selected were to indicate their understanding of compassion by selecting one of three definitions of compassion provided by the researchers. The study reported that, 60% of participants defined compassion as a “deep awareness of the suffering of others and a wish to alleviate it”; 28.2% selected “empathy and kindness” and only 9.3% selected the third option “a deep awareness of the suffering of others”. While the majority of participants felt that compassion was important (27.6%) or very important (69.6%); was influenced by a number of factors including culture (30.5%) [42], details about these factors were also limited in this study [41]. In addition, this study [42] did not delineate the differences in understandings of compassion between countries and the ethnic background of participants. In another qualitative study of Thai Buddhist family caregivers in Thailand investigating their experiences of caring for mentally ill patients, Sethabouppha et al. described that caregiving was a core Buddhist belief in general, with compassion being identified as the essence of Buddhist caregiving [43]. Using Buddhist principles, the authors defined compassion as “the deep feeling of sharing, giving aid, supporting, or showing mercy” (Page 48); and conceptualized caregivers’ compassion as being embedded in the Buddhist concepts of caring (*metta*) and support (*karuna*) [43].

Authors in other studies, while not directly defining compassion, have also tried to explore ethnically diverse perspectives related to specific domains of compassion, but not the entire construct [38, 44]. In a qualitative study comprised of Iranian subjects who were predominantly nurses, along with a few patients, participants described compassion as being expressed by non-verbal behaviours such as a smile, actively listening to patients, and touching and shaking hands with the patients [38]. In this study, touching and shaking hands were only deemed appropriate when caring for patients of the same sex because touching patients of a different sex was considered inappropriate and prohibited by Islam [38]. While appropriate bedside manners were also described as a way to demonstrate compassion, being indifferent towards patients and families, and using aggressive behaviours with them were described as non-compassionate actions within this particular ethnic group [38]. Touch was also identified as a primary way to convey compassion by African American participants in a thematic analysis study exploring their experiences about end-of-life care [44].

In addition to studies that focused patient and/or healthcare providers perspectives on the construct of compassion or a particular domain as a specific study aim, authors in a number of studies used compassion as a descriptor in interpreting the results of their study [45–49], even though participants were not asked specific questions pertaining to compassion, nor did they not mention compassion specifically in their responses. In a qualitative study exploring the meaning of good nursing care among last-year undergraduate nursing students in Thailand, compassion was reported as one of the key pillars of good nursing care [45]. Although the participants in this study were not asked to define compassion, nor did they describe compassion itself as important to patient care; the authors collated and interpreted participant responses related to giving care from the heart, having sympathy, being kind and helpful, having and sharing feelings, being friendly and being concerned, and being honest under the category of compassion [45]. Msiska et al., in a qualitative study, explored nursing students’ experiences of caring for HIV patients in the small sub-Saharan African country of Malawi [46], describing the provision of compassion to HIV patients as requiring HCPs to avoid prejudice and stigma associated with HIV, and creating an emotional connection with patients [46]. In another qualitative, authors examined the perspectives and experiences of caring among nurses in Hong Kong according to the 5Cs model (compassion, competence, confidence, conscience and commitment) [47, 50]. Study authors ascertained facets of compassion based on participant responses, including understanding patient needs, feelings and behaviours; showing empathy and sympathy; being efficient; meeting patient needs; being considerate; making allowances for patients and their families; flexibility in visiting hours; and giving simple explanation about procedure to ease patient's anxiety [47].

In a qualitative study of ethnically diverse parents (Hispanic, black, and white) who had lost a child in intensive care units in the United States, Brooten et al. [48] described how HCPs compassionate actions were perceived as helpful by parents. Using a pre-determined definition of compassion, the authors classified some of the participant responses as ‘compassionate actions’ which included: HCPs caring for the child as their own, informing parents when their child was nearing death so that they could hold their child; non-verbal expressions of compassion towards the parents such as a smile, hug or providing a beverage; maintaining a hopeful perspective in spite of the incurable nature of the disease; communicating with parents in a language and at a level they could best receive; follow-up phone calls to the parents after the death of a child; crying and/or praying with parents after their child’s death; and attending the child’s funeral [48]. These ‘compassionate actions’ were deemed helpful by parents from all ethnicities, although the relationship between the HCPs’ ethnic background and parents’ ethnic background was not examined [48].

In a commentary focused on cultivating compassion across different ethnicities and cultures, the author drew on their personal experience as a patient, noting that although compassion is considered a virtue in most major religions and philosophies, its antecedents and how it is expressed across ethnic backgrounds likely varies [49]. The author suggests that it is likely easier for nurses to express compassion toward ‘Western’ patients as they are typically more direct in expressing and sharing their feelings with the nurses. In contrast, ‘Eastern’ patients were described as less direct and less open to sharing their feelings, making it difficult for nurses to discern patients’ needs and making both patients and nurses feel awkward when feelings where addressed in a direct manner. Despite these differences between ‘Eastern’ and ‘Western’ patients, the author, who worked as a nurse in both Korea and the United States believed that these challenges to providing compassion nonetheless could be overcome, primarily through acts of human caring which included touching, listening, and acknowledging and relieving patients’ pain and suffering [49].

### Perceptions of facilitators and barriers to compassion within ethnic groups

Two studies explored factors which could act as facilitators or barriers of compassionate care within diverse ethnic groups [51, 52]. The first study was a qualitative study conducted in Iran exploring nurses’ perspectives on workplace and organizational barriers to compassionate care [52]. The study described an unsupportive organizational culture which included an excessive workload for HCPs, inadequate staffing resulting in a further increase in workload, and a lack of emphasis on compassionate care as barriers to HCP compassion within this context. The study highlighted that providing compassion in this context was contingent on a collective responsibility owned and shared by organizations, rather than individual responsibility of HCPs to provide compassion [52]. While representing barriers to compassion, the study did not discuss ethnic factors specifically or provide evidence on whether and how these generalized barriers within healthcare are specific or particularly detrimental to compassionate care.

A second study, conducted by the same Iranian research team aimed to explore the facilitators of compassionate care from nurses’ perspectives [51]. Consistent with their previous study [52], positive role models of compassion during training and their practice setting were one of the key facilitators [51]. Additionally, the study highlighted personal qualities and experiences that functioned as facilitators of HCPs’ compassion, namely: personal values, positive family upbringing, and personal faith/beliefs. In addition, personal experiences of suffering were described as facilitators of compassion, as HCP were able to relate to patient’s suffering and to understand the impact that compassion had in alleviating it [51]. While both these studies [51, 52] were conducted exclusively with Iranian nurses, it was not clear if the facilitators and barriers described in these studies were specific to Iranian nurses, or to the nursing profession in general.

### Perspectives on the importance and impact of providing compassion to ethnically diverse patients

A number of studies discussed the importance and impact of compassion when caring for patients from ethnically diverse backgrounds [53–55]. A qualitative study examined the hospitalization experience of the members of Mi’kmaq, a First Nation Community in New Brunswick, Canada [53], noting that compassion and a non-discriminatory attitude by HCPs was essential in overcoming cultural differences and challenges, suggesting that these qualities may create effective cross-cultural relations by actually compensating for a lack of HCP’s knowledge about their patient’s culture [53]. A separate qualitative study consisting of a sample of multi-ethnic patients explored perceptions of the significance of ethnicity in therapy relationships when the HCP was of a different ethnicity [54]. The majority of participants were apprehensive to discuss ethnic/culture issues with a therapist from a different ethnic background as they perceived those therapists as being unable to relate to their experience living as an ethnic minority, thereby hindering the development of a deep and emotional connection [54]. Again, despite acknowledging these culture related differences as barriers, participants felt that these barriers could nonetheless be mitigated through a therapist who demonstrated compassion in genuinely and sensitively seeking to understand these culture related issues [54]. Finally, a qualitative study describing HCP characteristics desired by African-American patients in prenatal care in the US reported that compassionate care was one of the most important traits that patients felt their HCP should possess [55]. The authors, who defined compassionate care as making the patients feel comfortable and not judged, felt that compassion was particularly important and served a medium in facilitating the disclosure of sensitive health issues that may not otherwise be shared with their HCPs, which the authors suggested were due to traditional mistrust of the healthcare system among African-Americans [55]. As such, while these studies did not provide details on what constitutes compassion specifically, they each highlighted the considerable role that compassion can play in the provision of cross-cultural care.

Two studies explored if compassion related outcomes varied among patients of different ethnicities [56, 57]. A quantitative study examined African-American, Latinos and White patients’ satisfaction with their physicians and healthcare [56]. Physicians who demonstrated a compassionate and respectful style were associated with increased patient satisfaction with their physicians across all ethnic groups [56]. However, an association between higher physician compassion and satisfaction with their healthcare experience was only observed among Whites and English-speaking Latinos [56]. In a separate quantitative study [57], authors explored the associations between physician’s traits and patients’ (White, Asian, African-Americans and Hispanics) quality of life. Physician compassion, which the authors defined as “expressing concern for patients’ feelings and being respectful of patient as a person” (Page 582); was reported as one of the factors associated with higher quality of life (QoL) among African-American, Hispanic and White patients, while no significant associations were reported for Asian patients [57].

A number of studies investigated compassion related outcomes, while utilizing a mixed sample of Western and ethnically diverse patients, did not investigate or identify differences between ethnic groups [44, 48, 58, 59]. In a qualitative study of non-English speaking patients admitted to an Australian hospital who spoke Arabic, Italian, Vietnamese, Chinese, Croatian, Serbian and Spanish, reported that being treated compassionately was one of the most important HCP trait desired by the patients in acute care [58]. The construct of compassion and the traits associated with it, however were not defined or explored in this study, with compassion being employed as an overarching term to classify HCP qualities of kindness and respect within routine care [58]. Highly compassionate HCPs were also described as key facilitators in helping ethnically diverse parents of children who died in a US intensive care unit cope with their loss [48]. A systematic review by Bosma et al. described that ethnic minorities in Western countries desire compassion in hospice and describe an ideal hospice HCP as an individual who displays compassionate qualities [59]. The importance of compassion to ethnic minorities in comparison to Westerners was unclear, as the review concluded that the expectations of patients in hospice related to compassion are similar across ethnic and cultural groups [59]. Jenkins et al. also emphasized the importance of compassion in the end-of-life care of African American patients and their family members; however, details on what compassion specifically entailed or its effects were not provided [44].

While most studies identified compassion related outcomes among ethnic minorities living in Western countries, few studies examined compassion related outcomes among ethnically diverse participants living in their home countries [60–63]. Fox et al. [60] published a narrative of 5 case studies, with one case study focused on the importance of compassion in end-of-life care, documenting the author’s experience of observing a patient coordinator within a palliative care unit in India. The authors described that the patient coordinator’s acts of compassion (soothing touch and a soft voice) alleviated distress during the last stages of a patient’s life [60]. The authors also felt that the patient coordinator’s soothing touch was as salient as the medications prescribed by the doctor, noting that it also had an ability to transcend culture and language barriers [60]. In a qualitative study with patients in Iran exploring factors that influence, promote, or compromise patient dignity; friendly and affable manners of the HCP, understanding patients’ emotions, and being patient were identified as important and categorized as ‘compassionate behaviours’ by the authors [61]. The authors went on to summate that these ‘compassionate behaviours’ seemed to positively influence patient’s sense of dignity and comfort, although no cause-effect relation was established [61]. In another qualitative study in an adult intensive care unit in Thailand with nurses caring for intubated patients who were unable to communicate verbally with their HCP [62], nurses mentioned that ‘compassionate understanding’ of patients’ situation was critical to understanding their healthcare needs and to enhancing patient care [62]. A separate qualitative study with palliative care nurses in Japan also suggested that patients’ distress and suffering could be ameliorated by nurses’ compassion, although the study provided no direct evidence between reduced patient suffering and nurses’ compassion [63].

## Discussion

We have synthesized the current literature focused on providing compassion within ethnically diverse patient populations, from the perspectives of patients and healthcare providers. Most studies used a pre-determined definition of compassion or introduced the term during the analysis phase based on researchers’ interpretation of study findings rather than asking ethnically diverse participants directly about their understanding of ‘compassion’. As a result, the literature related to ethnic perspectives of compassion in healthcare, is not only nascent, but nebulous, with conceptualizations of compassion ranging from a consortium of virtues (honesty, kindness, helpful, non-judgment) to a family of feelings within the provider that may or may not be expressed to the patient in a variety of ways [38, 41–44].

Despite this conceptual ambiguity, based on our synthesis of the limited number of studies focused on the perspectives, importance, experiences, and facilitators/barriers of compassion among diverse ethnic populations, it seems that compassion is universally recognized as a multi-faceted concept that is motivated by the virtues or personal qualities and beliefs of an individual in response to another persons’ suffering, through the provision of emotional support and physical acts to help the person in need. For example, a number of studies identified similar behaviours associated with compassion, such as caring touch that aimed to alleviate suffering [38, 44, 49, 60], echoing the results of studies of western patient populations which have identified similar traits associated with compassion [21, 27, 64]. A surprising finding, was that the importance of religious and spiritual guidance and practices as a source of compassion was only identified in one HCP study [43], even though religiosity and spirituality are of increased importance among ethnically diverse communities [65–67], and have been associated with compassionate care [68–70].

While at a conceptual level, compassion seems to be a universal construct that transcends ethnicities and cultures, ethnicity and culture seem to primarily influence how compassion is expressed and experienced in practice rather than how it is understood. For example, conveying compassion through supportive touch seems to traverse ethnicities and cultures [38, 44, 49, 60], however, for patients of Iranian ethnicities for example, supportive touch needs to take into consideration underlying social and religious beliefs, lest the good intentions of a Western HCPs be deemed uncompassionate by members of this ethnic group [38]. Patients’ ethnic background also seem to impact HCPs expression of compassionate care, with HCPs potentially expressing less affective compassion towards Korean patients, for example, due to a belief that patients from this ethnic group are less receptive to expressive feelings [49]. On the other hand, many Korean study patients preferred to be seen by a HCP from a different ethnic background because of fear of judgment from their fellow Korean HCP [54], thereby suggesting that patient-healthcare provider ethnicity concordance is not necessarily associated with enhanced compassionate care affirming the results of other studies [71]. Collectively, these results suggests that ethnic and cultural differences not only affect patients’ experiences of receiving compassion, but could also result in variance in HCPs’ provision of compassion—modifying their compassion based on the beliefs and assumptions they hold related to the ethnic background of the patient in their care. In addition to reflecting on their beliefs and assumptions, HCPs need to be mindful that while patients’ understanding and experiences of compassion vary by ethnic background, they also vary within ethnic groups. In doing so, this guards against a stereotypical approach that inadvertently results in healthcare providers expressing or muting certain facets of compassion based on false pretenses [33, 72].

Ultimately, this review suggests that compassion also has a therapeutic effect, serving as a universal care medium in cross-cultural care [48, 53–63]. Communication itself has been identified as an important medium for the delivery of compassion in healthcare [27], with communication barriers being a partiucular issue for ethnically diverse patients perceptions of quality care [37]. As such, approaching patients in a compassionate manner can have a bolus effect on cultural barriers it doesn’t serve as a panacea, requiring healthcare providers to actively counteract communication barriers in order for compassion to have an optimal therapeutic effect. The impact of compassionate communication on patients are modest, with HCPs who are perceived as being compassionate being more likely to elicit sensitive health information from ethnically diverse patients [55] and to develop a trusting therapeutic relationships among ethnic minorities that mistrust the healthcare system [9, 73–77]. While patient perspectives suggest that the way that care is provided may be as important as developing substantive cultural competieinces, [53, 54, 78, 79], this was not reported finding in any of the HCP studies reviewed reported similar findings [53–55]. Thus, additonal research needs to be conducted to examine HCPs perspectives on the role and impact that compassion has in overcoming cultural communication barriers.

On another cautionary note, while the intent of this review was to recognize diversity in expressions and experiences of compassion based on ethnicity, adopting an overly prescriptive and stereotypical approach that generalizes an entire ethnic groups understanding and experience of compassion, may actually further compound the core issue—namely, that while patient’s ethnicity can provides a framework for providing culturally sensitive compassion, experiences of compassion are ultimately defined by individual members of these ethnic and cultural groups [56]. This review, cautions against a one size fits all approach across ethnic and within ethnic groups, emphasizing that perhaps the most essential domain of compassion, particularly for HCPs who have a different ethnic background than their patient, is seeking to understand, not only their ethnic influencers of compassion, but the individual. A recent study examining Canadian HCPs’ perspectives of compassion described that intentional acts of compassion by HCPs can help overcome these challenges [80]. HCPs of diverse ethnic backgrounds also feel that compassion is an essential component of good care and can be taught [42, 45]. Therefore, teaching the importance of compassion to HCPs along with cultural influencers effecting patients experience of compassion can help HCPs tailor and individualize compassion to the ethnically diverse patients they serve.

There were four notable limitations with the selected studies in this review. First, while the studies in this review focused on various ethnic groups, the generalizability and applicability of these findings to these ethnic groups as a whole was not determined [48, 55, 58, 61–63]. Second, this review included studies that occurred in ethnically diverse countries and ethnic minorities living in Western countries [38, 43, 45–47, 51, 52, 61–63], further effecting the generalizability of the findings as the results may be not be strictly due to ethnicity but the countries in which studies were conducted. A further limitation of this review was that only studies written in English were included in this review. By excluding non-English studies, we may have inadvertently missed important studies, particularly those with ethnically diverse participants in their home countries which were not published in English. Finally, we did not search the grey literature related to perceptions of compassion in healthcare from various ethnic backgrounds, which while not being an academic source of knowledge, may in fact hold significant information and insights about the topic.

## Conclusions

In this narrative systematic review, we have described the perspectives, importance, experiences, and facilitators/barriers of compassion within healthcare among diverse ethnic patient populations. While an emerging evidence base has been established, there is a need for further research in this field in general, including more contextual studies to exam how the understanding, expression, delivery, and experiences of compassion can vary within these populations, within and outside of Western healthcare systems. As ethnic diversity continues to grow within Western countries, it is imperative to understand and adapt to the needs and experiences of different ethnic groups in order to provide ethnically and culturally sensitive compassionate care.

## Supporting information

S1 Table PRISMA checklist. (PDF)
